# Temperature increase significantly enhances nociceptive responses of C-fibers to ATP, high K^+^, and acidic pH in mice

**DOI:** 10.3389/fncel.2023.1131643

**Published:** 2023-02-09

**Authors:** Yurii Tkachenko, Volodymyr Khmyz, Dmytro Isaev, Oleksandr Maximyuk, Oleg Krishtal

**Affiliations:** Bogomoletz Institute of Physiology, National Academy of Sciences of Ukraine, Kyiv, Ukraine

**Keywords:** ATP, pain, polymodal C-fibers, potassium ions, single-fiber recording, temperature dependence, nociception

## Abstract

It is well established that temperature affects the functioning of almost all biomolecules and, consequently, all cellular functions. Here, we show how temperature variations within a physiological range affect primary afferents’ spontaneous activity in response to chemical nociceptive stimulation. An *ex vivo* mouse hind limb skin-saphenous nerve preparation was used to study the temperature dependence of single C-mechanoheat (C-MH) fibers’ spontaneous activity. Nociceptive fibers showed a basal spike frequency of 0.097 ± 0.013 Hz in control conditions (30°C). Non-surprisingly, this activity decreased at 20°C and increased at 40°C, showing moderate temperature dependence with Q_10_∼2.01. The fibers’ conduction velocity was also temperature-dependent, with an apparent Q_10_ of 1.38. Both Q_10_ for spike frequency and conduction velocity were found to be in good correspondence with an apparent Q_10_ for ion channels gating. Then we examined the temperature dependence of nociceptor responses to high K^+^, ATP, and H^+^. Receptive fields of nociceptors were superfused with solutions containing 10.8 mM K^+^, 200 μM ATP, and H^+^ (pH 6.7) at three different temperatures: 20, 30, and 40°C. We found that at 30 and 20°C, all the examined fibers were sensitive to K^+^, but not to ATP or H^+^. At 20°C, only 53% of fibers were responsible for ATP; increasing the temperature to 40°C resulted in 100% of sensitive fibers. Moreover, at 20°C, all observed fibers were silent to pH, but at 40°C, this number was gradually increased to 87.9%. We have found that the temperature increase from 20 to 30°C significantly facilitated responses to ATP (Q_10_∼3.11) and H^+^ (Q_10_∼3.25), leaving high K^+^ virtually untouched (Q_10_∼1.88 vs. 2.01 in control conditions). These data suggest a possible role of P2X receptors in coding the intensity of non-noxious thermal stimuli.

## Introduction

The signs of inflammation among others include edema, fever, erythema, acidosis, pain, and hyperalgesia (Marchand et al., 2005; Casimir et al., 2018). Possible candidates for the algogenic factors released from the cytosol are potassium ions, hydrogen ions, and ATP (Amaya et al., 2013). Nociceptors are peripheral primary sensory neurons that first detect chemical, mechanical, and thermal pain stimuli.

The C-polymodal nociceptors (CPNs) respond to a variety of noxious stimuli (i.e., mechanical, thermal, and chemical stimuli). Amongst the nociceptor population, C-mechanoheat fibers (C-MH or polymodal nociceptors) have been shown to be highly responsive to P2X agonists (Hamilton et al., 2001) and acidic pH (Kessler et al., 1992). The threshold levels for C-MH fibers were found to be in the range from pH 6.9 to 6.1, while the mean heat threshold was 40.7°C (the intracutaneous temperature at first spike discharge).

Tissue acidosis is the decisive factor for non-adapting nociceptor excitation that elicits sustained pain (Steen et al., 1992, 1996; Pan et al., 1999). TRPV1 and ASICs ion channels expressed in DRG neurons are extracellular acid sensors, contributing to acid-induced nociception within the physiological pH range (below pH 7.3 for ASICs, and 6.0 for TRPV1 at 35°C) (Caterina et al., 1997; Waldmann et al., 1997; Blanchard and Kellenberger, 2011). ATP released from damaged or inflamed tissues can act at P2X3 receptors expressed in primary afferent neurons. P2X3 are found in a subset of small-diameter, primary afferent neurons (North, 2004; Dosch et al., 2018). Increasing the K^+^ ion concentration in the vicinity of the nerve terminal depolarizes and activates nerve fibers and produces ongoing pain (Markowitz and Kim, 1992; Wanachantararak et al., 2011). Tissue cooling increases the pain threshold and has quantitatively different effects on axonal excitability (specifically, threshold, refractoriness, strength-duration time constant, rheobase, and nerve conduction velocity) (Kerschan-Schindl et al., 1998; Burke et al., 1999). Heating the skin to subthreshold heat pain temperatures potentiates the experienced acid pain in humans, on the other hand, cooling the skin caused abrupt pain relief (Jones et al., 2004).

Cold has also been shown to reduce pain effectively in the treatment of injuries and inflammation in medicine because it is cheap, fast-acting, non-toxic, and effective and has no risk of sensitization (Swenson et al., 1996; Dixit et al., 2013). Indeed, the cold is extensively used for pain relief since 3,500 B.C. up to date (Bierman, 1955), however, the scientific evidence from clinical trials is still very patchy (Ernst and Fialka, 1994). Here we present electrophysiological data on the effect of temperature changes on the excitability of saphenous nerve primary afferents in response to increased K^+^, ATP, and H^+^. We have found that the temperature increase significantly facilitates responses to the ATP and H^+^ with apparent Q_10_∼3.

## Materials and methods

### Animals

Adult (6–7 weeks old) male mice weighing 22–31 g were used in this study. Animals were obtained from the animal facility of Bogomoletz Institute of Physiology, housed on a 12-h light-dark schedule, and given food and water *ad libitum*. The following experimental procedures were performed in accordance with the recommendations in the guidelines of the Bogomoletz Institute Animal Care and Use Committee. All efforts were made to avoid or minimize suffering.

### Skin-nerve preparation

The skin-nerve preparation was used to record from single primary afferents, as previously described (Zimmermann et al., 2009). Under deep anesthesia (urethane, 2 g per kg, i.p.), the saphenous nerve and its innervating territory on the hairy hind paw skin were subcutaneously dissected and excised. After dissection, the animals were euthanized by an intraperitoneal injection of a lethal dose of urethane. Next, the skin was gently stretched and pinned corium side up in the organ bath for pharmacological application to the receptive fields of single sensory units. The end of the nerve was gently threaded through a hole into a separate recording chamber for fiber teasing and single-unit recording. The tissue and recording chamber were separately superfused with a modified Krebs–Henseleit solution (in mM): NaCl 118, KCl 5.4, NaH_2_PO_4_ 1.0, MgSO_4_ 1.2, CaCl_2_ 1.9, NaHCO_3_ 25.0, and dextrose 11.1, and gassed with 95% O_2_–5% CO_2_, pH 7.4, at a flow rate of 8 and 3 ml min^–1^, respectively. The temperature in the organ bath and recording chamber was adjusted and maintained at 20, 30, and 40°C ± 0.1°C. Temperature control was carried out by a digital thermostat with a miniature thermocouple.

### Extracellular recordings of action potentials

The nerve was placed on a small mirror in the recording chamber. Filaments were teased from the desheathed nerve by using sharpened watchmakers’ forceps. Small filaments were teased out of the whole nerve and subdivided under microscopic control. Extracellular recordings were performed by using a borosilicate glass (OD 1.5 mm, ID 0.86 mm) suction electrode (pulled with a Flaming−Brown micropipette puller, Model P-97, Sutter Instrument CO., Novato, CA, USA). The electrode was placed into an electrode holder connected to a low-noise differential amplifier and connected to a 5 ml syringe for applying negative (“suction”) pressure. A reference electrode of silver/silver chloride wire and an earthed silver/silver chloride pellet were placed in the perfusion fluid of the recording chamber. Single nerve fiber activity was amplified and filtered out (low cut-off, 0.3 kHz; high cut-off, 1 kHz) by using an AC/DC differential amplifier (Model 3000, A-M Systems, Inc., Carlsborg, WA, USA). The amplified signal was then recorded to a PC *via* a Digidata 1200 analog-to-digital converter (Axon Instruments, USA) by using WinEDR v3.9.1 and WinWCP V4.5.4 Software (Dr. John Dempster, University of Strathclyde, UK). The innervation of the skin was tested with a blunt glass rod mechanically searching for the receptive held of a nerve fiber (Kress et al., 1992). An analog stimulus isolator (Model 2200, A-M Systems, Inc., USA) was used to electrically stimulate the receptive field with a fine steel electrode to measure conduction latency in milliseconds, which was then used to estimate conduction velocity. Recordings were obtained only from single fibers that could be easily discriminated according to amplitude and shape. Latency measurements were used to determine precise cutoff points among the three major afferent fiber types Aβ, Aδ, and C. Sensory neurons with slow conduction velocities, generally less than 10 m/s, were considered to be Aδ fibers, while faster neurons were Aβ fibers. A cutoff of 1.0 m/s was used to distinguish between myelinated and unmyelinated fibers. Recorded single fiber activities were analyzed offline by using the template matching function of Spike 2 software (CED, Cambridge, UK). Activation energy Ea was calculated from the frequency of the spikes (*f*) at a given temperature T (K) by using the Arrhenius plot of ln(f) vs. 1/T. The Q_10_ was calculated from the equation:


$$Q_{10}=\frac{10\,E_{a}}{e^{R\,T\,\left(T+10\right)}}$$

where *R* = 8.3114 J mol^–1^ K^–1^ is the universal gas constant.

The Q_10_ for the conduction velocity was calculated from the equation:


$$Q_{10}={\left(\frac{X_{2}}{X_{1}}\right)}^{10/\left(T_{2}-T_{1}\right)}$$

### Statistical tests

All statistical tests were carried out by using Origin Pro 8.5 (Origin Lab. Corp., Northampton, MA, USA). All values are given as mean ± standard error of the mean (SEM). Statistical analysis of the differences between nerve activities was assessed by one-way repeated measures ANOVA with Bonferroni *post-hoc* test. *P*-values less than 0.001 are reported as *p* < 0.001.

### Chemical stimulation

The receptive field of each nerve fiber was sequentially stimulated by one of three chemical stimuli (H^+^, K^+^, or ATP) at three temperatures (20, 30, and 40°C). For stimulation with H^+^ receptive field of nerve fiber was superfused with low bicarbonate buffer of the following composition (mM): NaCl, 142; KCl, 5.4; NaH_2_PO4, 1.0; MgSO_4_, 1.2; CaCl_2_, 1.9; NaHCO_3_, 1.0; and dextrose, 11.1. The pH of the low bicarbonate buffer solution was adjusted to 6.7 by saturation with carbogen gas (95% oxygen, 5% CO_2_) (Kollarik and Undem, 2002). K^+^ was used at a concentration of 10.8 mM to keep the osmolarity of the modified Krebs bicarbonate buffer solution constant, and Na^+^ was adjusted accordingly. ATP was added to the Krebs bicarbonate buffer solution at a concentration of 200 μM. Each chemical stimulation was followed by a washout period lasting more than 5 min. A nerve fiber was considered “activated” by chemical stimulation when the instant spike frequency (the number of spikes per s.) in response to the application of K^+^, H^+^, or ATP was increased by at least 50% in comparison with the activity in the control. Fibers whose activity did not return to the control level once the stimulus was removed were not taken into account.

### Drugs and chemicals

All chemicals used were purchased from Sigma-Aldrich (St. Louis, MO, USA), VWR (Radnor, PA, USA), or Carl Roth (Karlsruhe, Germany).

## Results

### General characteristics

**FIGURE 1:**
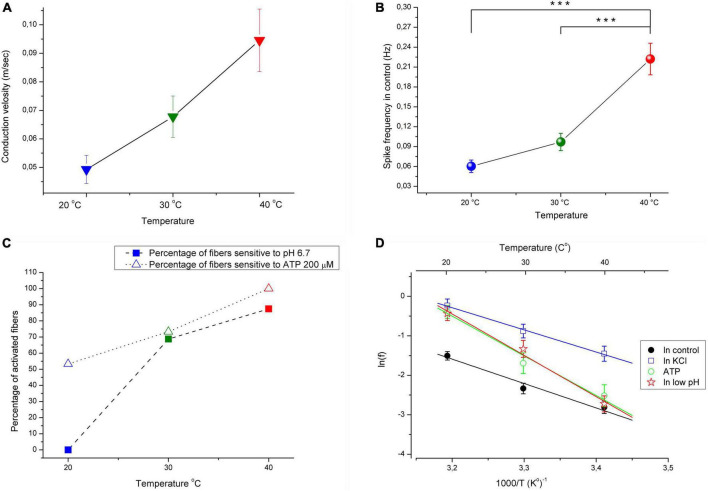
**(A)** Conduction velocity is temperature dependent with Q_10_ ≈ 1.4. **(B)** Increasing the temperature under control conditions from 30 to 40 °C resulted in significant increases in the spontaneous activity of afferent nerve fibers (*n* = 71; MEAN ± SEM; *p* < 0.001). **(C)** The increasing temperature during application of low pH solution (pH 6.7) and ATP 200 μM results in increasing the number of fibers activated by low pH and ATP. KCl 10.8 mM activated 100% nerve fibers at all investigated temperatures (not showed). **(D)** Arrhenius plot of the temperature dependence of the spikes frequency of CMH nociceptors in control–black closed circles and line; during acidification–red open stars and line; during application ATP (200 μM)–green open circles and line; during stimulation fibers with high KCl (10.8 mM)–blue open squares and line. Here, averaged data (MEAN ± SEM) over the entire range of chemical stimulation and control recording were taken into account. The activation energy Ea and Q_10_ were determined from linear regression parameters (see section “Materials and methods” for details). ***p < 0.001.

**FIGURE 2:**
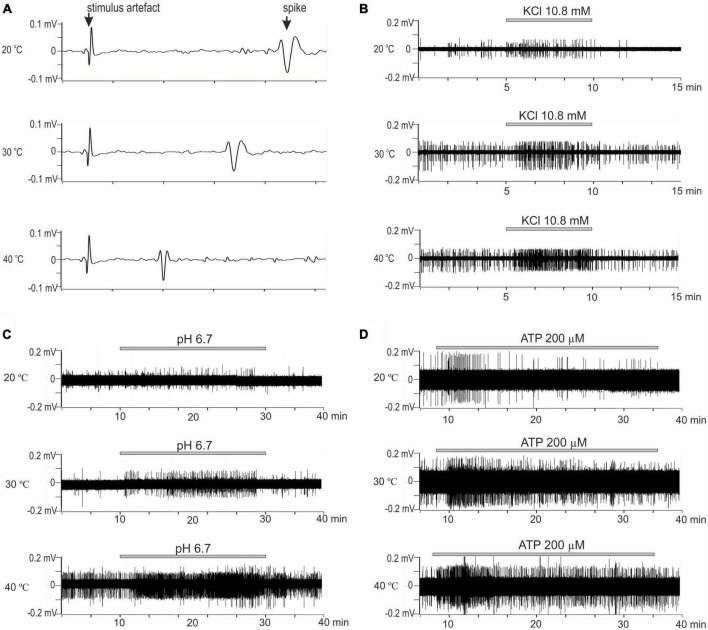
**(A)** Representative example recording of activity from the skin CMH-afferents. Three consecutive recordings from a C-fiber in response to electrical stimulation at 20, 30, and 40°C. Sweeps show the change in the conduction velocity and waveform of the action potential with a temperature change. Increasing temperature results increase in conduction. Arrow indicates artifact from electrical stimulation and action potential; *x*-axis: delay in ms; *y*-axis: voltage signal amplitude. **(B–D)** The original recordings showed responses of nerve filaments with CMH-fibers to chemical stimulation by irritants at 20, 30, and 40°C. **(B)** Stimulation by high K^+^ (10.8 mM) for 5 min. **(C)** Stimulation by low pH solution (pH 6.7) for 20 min. **(D)** Stimulation by ATP 200 μM for 20 min (25 min of control recordings before and after ATP application not shown).

A total of 71 C-MH single fibers innervating the hind paw glabrous skin from 36 mice were studied. Fibers that did not develop ongoing activity during or after the initial characterization of their sensory properties were included in the study. All tested C-fibers had conduction velocities below 1.0 m/s at 30°C (see section “Materials and methods”). The present study aimed to examine the effect of temperature within physiological limits on the activity of pain receptors in mouse skin. We examined the effect of algogenic factors (increased ATP, K^+^, and H^+^) on the activity of single C-fibers from the Saphenous nerve at various temperatures in the physiological range of 20, 30, and 40°C. Increasing the temperature under normal conditions resulted in a statistically significant increase in spontaneous basal activity of tested nerve fibers from 0.097 ± 0.013 Hz at 30°C to 0.22 ± 0.024 Hz at 40°C (Figure 1B; *n* = 71, *p* < 0.001). Decreasing the temperature to 20°C resulted in the opposite effect: the activity was decreased to 0.06 ± 0.009 Hz (*p* = 0.18). The mean conduction velocity of the unmyelinated afferent fibers was 0.067 ± 0.007 m/s at 30°C, 0.049 ± 0.005 m/s at 20°C and 0.095 ± 0.011 m/s at 40°C (Figures 1A, 2A; *n* = 71).

### Lowering pH

In this experimental group was investigated 33 skin C-MH nerve afferents. Low bicarbonate buffer solution (pH 6.7) was repeatedly superfused over the receptive fields at 20, 30, and 40°C for 20 min at 10 min intervals.

**FIGURE 3:**
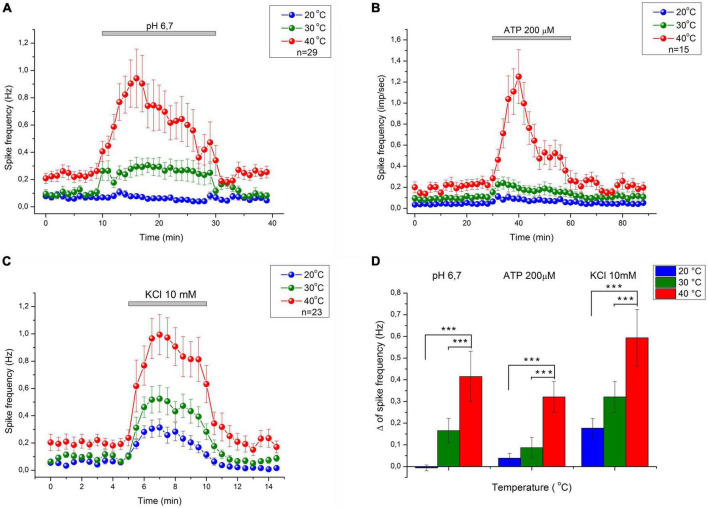
The average response of CMH nociceptors to low bicarbonate buffer solution (pH = 6.7) **(A)**, ATP 200 μM **(B)**, and K^+^ 10.8 mM **(C)** in mice skin-nerve preparation at 20, 30, and 40°C. C-fibers nociceptors displayed an increasing response to nociceptive agents: ATP, low pH, and high K^+^ concentration with increasing temperature. The value of spikes frequency in response to high K^+^, low pH, and ATP, was highest at 40°C and lowest at 20°C. **(D)** Difference between MEAN of spikes frequency during chemical stimulation and control recording–Δ (delta) at 20, 30, and 40°C. ***p < 0.001.

C-mechanoheat fibers showed a sparse (0.07 Hz) and irregular baseline discharge or were silent under control conditions at the beginning of the experiment at 20°C (pH 7.4). Administration of a solution with a pH of 6.7 did not change the average firing frequency of nerve afferents (*n* = 29; *p* = 1). An increase in temperature to 30°C led to an increase in the frequency of spontaneous activity to 0.099 ± 0.025 Hz under control conditions. The administration of pH 6.7 solution increased the spike frequency of skin nerve afferents to 0.265 ± 0.056 Hz (*n* = 29; *p* = 0.334) and activated 69.7 % (23/33) fibers. In control at 40°C, nerve fiber activity increased to 0.235 ± 0.041 Hz (Figures 2C, 3A). Low bicarbonate buffer application at 40°C resulted in significant growth spike discharge frequency in primary afferents to 0.65 ± 0.117 Hz (Figure 3D; *n* = 29, *p* < 0.001) and activated 87.9% (29/33) of nerve afferents (Figure 1C). The activity under low pH was also significantly higher at 40°C compared to their activity at 30°C (*p* = 0.00047) and compared to activity at 20°C (*p* < 0.001; Figure 3D). The value of spikes frequency was highest at 40°C and lowest at 20°C. An increase in temperature also has increased the number of nerve fibers activated by low pH.

### ATP 200 μM

In this experimental group, the activity of 15 cutaneous afferents was investigated. The receptive fields of sensory endings were tested using successive applications of ATP 200 μM for 30 min at 60 min intervals at three different temperatures (20, 30, and 40°C). Activation of nerve afferents by ATP, and their activity under ATP application and control conditions also showed positive temperature dependence. At 20°C, ATP activated 53% (8/15) of nerve fibers and slightly but statistically insignificant increased nerve activity from 0.04 ± 0.013 Hz under control conditions to 0.08 ± 0.02 Hz during ATP application (*n* = 15; *p* = 1). At 30°C, ATP activated 73.3% (12/15) of nerve fibers and also induces slight but statistically insignificant increase in nerve activity from 0.097 ± 0.024 Hz under control conditions to 0.18 ± 0.05 Hz during ATP application (*n* = 15; *p* = 1). At 40°C (Figures 2D, 3B), ATP activated 100% (15/15; Figure 1C) of nerve fibers and increased nerve activity from 0.2 ± 0.034 Hz under control conditions to 0.67 ± 0.11 Hz during ATP application (*n* = 15, *p* < 0.001). The activity under ATP 200 μM was also significantly higher at 40°C compared to their activity at 30°C (*p* < 0.001) and compared to activity at 20°C (*p* < 0.001; Figure 3D). Thus, an increase in temperature causes an increase in the activity of nerve fibers and their sensitivity to ATP.

### KCl 10.8 mM

Twenty-three CMH-nerve fibers were investigated. Administration of 10.8 mM KCl to the receptive field has activated all nerve afferents (100%, 23/23) at all tested temperatures (Figure 2B). Superfusion with high K^+^ solution resulted in growth spike discharge frequency primary afferents compared to control: from 0.058 ± 0.013 to 0.234 ± 0.045 Hz at 20°C (*n* = 23; *p* = 0.195). A statistically significant increase in spike discharge frequency impulses compared with the control was observed at 30 and 40°C: from 0.094 ± 0.017 to 0.415 ± 0.072 Hz at 30°C (*n* = 23, *p* = 0.000176); and from 0.199 ± 0.044 to 0.793 ± 0.13 Hz at 40°C (*p* < 0.001) (Figure 3C). The activity under high K^+^ was also significantly higher at 40 C compared to their activity at 30°C (*p* < 0.001) and compared to activity at 20°C (*p* < 0.001; Figure 3D). The activation of nerve fibers by potassium was positively temperature-dependent.

### Q_10_ and activation energy

The Arrhenius plot of spikes frequency of C-MH nociceptors demonstrates the temperature dependence for the frequency of the spikes within the range of 20–40°C (Figure 1D; *n* = 71). Generation of action potentials requires considerable energy of activation calculated from the Arrhenius plot (see section “Materials and methods”). The values of Ea (in kJ/M) were: 51.4 ± 8.6 for basal activity, 46.7 ± 2.5 for K^+^, 83.6 ± 10.6 for ATP, and 87.1 ± 8.6 for low pH. Corresponding Q_10_ for temperature-dependence of spikes frequency (*f*) between 20 and 30°C were: 2.01 ± 0.2 for basal activity, 1.88 ± 0.06 for K^+^, 3.11 ± 0.44 for ATP, and 3.25 ± 0.38 for low pH. The Q_10_ values for the spike frequency between 30 and 40°C were 1.92 ± 0.21 for basal activity, 1.81 ± 0.06 for K^+^, 2.89 ± 0.39 for ATP, and 3.02 ± 0.33 for low pH, respectively. The values of the Q_10_ were for conduction velocity: 1.38 ± 0.023 between 20 and 30°C, and 1.4 ± 0.034 between 30 and 40°C.

## Discussion

Temperature affects the function of virtually all biomolecules, and hence, affects cellular metabolism. It has been known for many centuries that temperature changes within the physiological range strongly affect pain sensitivity, but the molecular mechanisms underlying this effect remain poorly understood. The temperature coefficients of fluxes in open channels are low (Q_10_ = 1.3–1.5) indicating that the enthalpy barriers to the flow are about as low as for the ordinary aqueous diffusion. For these and other reasons neurophysiologists usually describe ionic flow within the channels using integrated equations of electrodiffusion derived from the differential Nernst- Planck equations for free diffusion of ions in a viscous medium (Hille, 1975). In our experiments, the Q_10_ values for the basal activity in control (Q_10_∼2), the K^+^ -induced discharges (Q_10_∼1.9), as well as the nerve fibers conduction velocity (Q_10_∼1.4) coincide with these values. Such value of Q_10_ for the conduction velocity is in a good correspondence with previously published data (Lowitzsch et al., 1977; Berg-Johnsen and Langmoen, 1992; Leandri et al., 2008). This may indicate that the temperature dependence of these parameters is directly a consequence of the temperature dependence of the current through the sodium and potassium channels of the cell membrane.

Molecular basis of temperature sensation remains poorly understood. Transient receptor potential (TRP) superfamily exhibits a wide range of thermal activation thresholds (>43°C for TRPV1, >52°C for TRPV2, ≥34–38°C for TRPV3, ≥27–35°C for TRPV4, ≥25–28°C or TRPM8 and <17°C for TRPA1), and are expressed in primary sensory neurons as well as other tissues (Tominaga and Caterina, 2004). However, electrophysiological recordings from C-fibers in the murine skin-nerve preparation in trpv1-/-, trpm2-/-, and trpv1:trpa1:trpm3-/- mutant mice indicated that the ability of polymodal C-fibers to detect both warm and cooling stimuli was largely unchanged compared to wild type mice (Paricio-Montesinos et al., 2020). The aforementioned points to the possibility of additional molecular mechanisms contributing to thermal sensitivity.

Indeed, it has been established that the P2X3 receptors, which are thought to be responsible for nociception and are nearly exclusively expressed in mammalian sensory neurons, also contribute to the encoding of temperature perception (Souslova et al., 2000). The responsiveness of P2X3 receptors is strongly reduced at low temperatures, suggesting a role for these receptors in the analgesic effects of cooling. Homomeric P2X3 receptors desensitize rapidly and recover from desensitization very slowly (20–25 min at room temperature). The recovery from desensitization of P2X3 receptors is greatly accelerated with temperature increase (Q_10_∼10) (Khmyz et al., 2008). Unfortunately, the mysterious difference between Q_10_ for ATP-induced nerve excitation and recovery from P2X3 receptor desensitization remains unknown. Temperature insensitive P2X2 subunits in heteromeric P2X channels expressed on nerve endings could explain this. It has also been proposed that the G protein-coupled P2Y1 receptor plays a role in temperature sensitivity in cutaneous sensory afferents (Molliver et al., 2011). It was reported that ASICs but not TRPV1 channels are the principal pH sensors of DRG neurons at 35°C in the pH range ≥ 6 (Blanchard and Kellenberger, 2011). We used a buffer solution with a pH of 6.7 to rule out the involvement of TRPV channels in nerve endings’ response to low pH. Indeed, AMG-517, a selective antagonist of TRPV1 channels, did not produce any distinguishable changes to the observed thermal sensitivity of low pH-induced responses (data not shown). On the other hand, the effect of temperature-dependence of ASICs gating is quite weak showing Q_10_ values close to typical values for gating processes involving conformational changes of ion channels. Moreover, increasing the temperature accelerates the inactivation of ASIC channels expressed in DRG cells and, in turn, decreases the number of action potentials induced by acidification (Blanchard and Kellenberger, 2011), thus producing the opposite effect to that reported here for the nociceptive nerve endings. However, the acidification also accelerated the recovery of P2X3 receptors from the desensitized state (Maximyuk et al., 2007), so we cannot exclude that the thermal sensitivity of pH-induced responses could be mediated by P2X3 receptors rather than ASICs. In conclusion, the temperature dependence of ATP-induced responses of nociceptive nerve endings is a complex mechanism involving various types of purinergic receptors. The thermal sensitivity of H^+^-induced responses is far more enigmatic, necessitating further investigations.

Our data demonstrate that the increase in temperature from 30 to 40°C increases the basal activity of nociceptive afferents, whereas the decrease from 30 to 20°C attenuates this activity. These changes in the activity of afferents can be easily explained by classical temperature sensitivity of ionic fluxes through the channels in terms of Nernst- Planck equations for free diffusion of ions in a viscous medium (Hille, 1975). However, at the same time, the increase in the temperature within these limits significantly enhances (Q_10_∼3) the nociceptive response to chemical stimulation of C-fibers by ATP and acidic pH, but not to stimulation with high K^+^, which has temperature-dependence (Q_10_∼2) virtually the same as basal activity. Non-surprisingly, the decrease in temperature induces quantitatively with the same, but opposite effects. The higher values of the Q_10_ for the sensory nerve activity due to pH lowering or ATP application as compared with the corresponding Q_10_ in control and at high K^+^ indicate the possible role of proton-sensitive and ATP-sensitive receptors in in temperature perception.

Thus, an increase in temperature within physiological limits is a strong factor that reduces the threshold of excitation for the nociceptive nerve fibers in response to the chemical irritants, enhancing hyperalgesia and nociceptive response. The use of transgenic models and effective selective pain receptor blockers can shed light on these mechanisms.

## Data availability statement

The raw data supporting the conclusions of this article will be made available by the authors, without undue reservation.

## Ethics statement

The animal study was reviewed and approved by the Bogomoletz Institute of Physiology.

## Author contributions

YT: conducted experimental research, experimental data processing, and analysis of experimental data. VK: analysis and interpretation of experimental data. DI: substantial contributions to the conception and design of the work. OM: drafting the work, analysis, and interpretation of data for the work. OK: project management and critical review of the relevance of experimental research. All authors contributed to the article and approved the submitted version.
